# Proximity to water shapes the distribution of natural elephant mortality in Hwange National Park, Zimbabwe

**DOI:** 10.1038/s41598-025-19902-x

**Published:** 2025-10-21

**Authors:** Blessing Kavhu, Kudzai Shaun Mpakairi, Nobesuthu Ngwenya, Timothy Kuiper, Henry Ndaimani, Edson Gandiwa

**Affiliations:** 1 Environmental Studies, University of California Santa Cruz, Santa Cruz, CA USA; 2 Department of Ecology and Evolutionary Biology, University of California Santa Cruz, Santa Cruz, CA USA; 3 Department of Earth Sciences, Institute for Water Studies, University of the Western Cape, Cape Town, South Africa; 4 Scientific Services Unit, Zimbabwe Parks and Wildlife Management Authority, Hwange National Park, Hwange, Zimbabwe; 5 School of Natural Resource Science and Management, Nelson Mandela University, George Campus, George, South Africa; 6 International Fund for Animal Welfare (IFAW), Harare, Zimbabwe; 7 Scientific Services Unit, Zimbabwe Parks and Wildlife Management Authority, Headquarters, Harare, Zimbabwe; 8 Finnish Geospatial Research Institute, National Land Survey of Finland, Espoo, Finland

**Keywords:** African Elephants, Mortalities, Machine Learning, Environmental Variability, Drought, Zoology, Ecology, Environmental sciences

## Abstract

While elephant poaching has received considerable attention, natural mortality can at times surpass human-induced deaths, especially under environmental stress. Understanding the ecological drivers of natural elephant mortality is therefore crucial for informing reintroduction efforts and preventing mass die-offs. In this study, we investigated environmental predictors of natural elephant mortality in Hwange National Park, Zimbabwe, using mortality records from 2020 to 2022. We applied four machine learning species distribution models, Random Forest, Gradient Boosting, Maximum Entropy, and Extreme Gradient Boosting, along with their ensemble to model mortality hotspots. The ensemble model outperformed individual models, achieving a True Skill Statistic of 0.54 and a Receiver Operating Characteristic of 0.83. Among all predictors, distance to water sources was the most influential variable (accounting for > 55% of model importance), with most mortalities occurring within 6 km of water points. Other key predictors included climate water deficit, normalized difference vegetation index (NDVI), tree cover percentage, and elephant density (each contributing > 5%). In contrast, maximum temperature of the warmest month and elevation had minimal predictive power (< 4%). Our results provide actionable insights for conservation planning. Areas close to water sources, particularly during dry periods, should be prioritized for monitoring and veterinary intervention. Meanwhile, regions with historically low mortality prevalences may serve as safer sites for reintroduction. This spatially explicit framework can help reduce post-release losses and enhance the long-term success of elephant conservation initiatives, especially in the face of ongoing environmental change.

## Introduction

The geographic range of African elephants (*Loxodonta africana*) has contracted markedly across sub-Saharan Africa over the past two decades, primarily due to anthropogenic pressures such as poaching, habitat loss, and retaliatory killings resulting from human-elephant conflict ^1–6^. In addition to these well-documented threats, climatic and ecological factors, particularly the availability of food and water, also play a significant role in shaping elephant distribution and movement patterns ^7,8^. Although habitat loss and the spatial dynamics of human-induced threats have been extensively studied, recent evidence highlights less-explored causes of elephant mortality. Specifically, an increasing number of unexplained deaths attributed to thermal stress, disease, bacterial infections, and starvation have been reported across the continent ^9–11^. These emerging mortality patterns are particularly concerning in regions where elephant populations are already under pressure, underscoring the urgent need to better understand the environmental drivers of natural mortality.

Elephant movement across savanna ecosystems is inherently dynamic and closely aligned with seasonal fluctuations in resource availability. Elephants do not use space uniformly but instead adapt their movements based on the spatial and temporal distribution of forage, water, and shade ^12–14^. When local resources become scarce, elephants may be compelled to enter high-risk areas such as agricultural lands or poaching zones, where they exhibit behavioral adaptations such as faster, more linear movements aimed at minimizing exposure to threats ^15,16^. While such behavioral plasticity may aid short-term survival, it imposes substantial energetic demands that can contribute to long-term physiological stress ^16,17^. Although elephant movement in response to anthropogenic threats is relatively well understood, behavior in areas associated with natural mortality remains poorly characterized. This gap is further exacerbated by a lack of spatially explicit data on natural mortality hotspots, limiting our ability to assess how ecological stressors interact with elephant behavior and survival. Addressing this gap is crucial for proactive, spatially informed conservation planning.

A promising approach to understand the environmental determinants of natural mortality involves the use of carcass data from elephants presumed to have died from natural causes, typically those found with intact tusks and no visible signs of trauma or poisoning. The Monitoring the Illegal Killing of Elephants (MIKE) programme under CITES has facilitated the collection and consolidation of such data, providing an important foundation for research in data-scarce contexts, particularly across Africa^18^. In the absence of comprehensive veterinary infrastructure and necropsy information, mortality data collected through MIKE protocols and toolkits offer a practical, if imperfect, means of documenting natural deaths. When paired with environmental datasets, these mortality records can inform spatial models capable of predicting prevalence of natural mortalities across landscapes. Species distribution modeling (SDM) techniques, particularly machine learning approaches such as Maximum Entropy (MaxEnt), Random Forest (RF), and Gradient Boosting Machines (GBM), have proven effective in analogous applications, including modeling species ranges, habitat suitability, and anthropogenic threat zones ^19–21^. However, the predictive performance of individual algorithms can vary depending on ecological complexity, data resolution, and spatial scale. To address this limitation, ensemble modeling has emerged as a robust method to improve prediction accuracy and reliability ^20–22^. In elephant ecology, ensemble SDMs have been successfully applied to forecast species distributions, poaching risk, and climate change impacts ^23–26^. Despite their demonstrated utility, ensemble models remain underutilized in studying the environmental determinants of natural elephant mortality.

Currently, spatially explicit analyses of natural elephant mortality are rare. Most investigations rely on clinical or laboratory assessments of carcasses ^11,27–29^, which, while informative, offer limited insight into the broader ecological and climatic contexts in which these deaths occur. A spatial perspective is especially critical in the era of global environmental change, where changes in temperature, precipitation patterns, and vegetation dynamics may exacerbate existing pressures on elephant populations. Understanding how these variables interact to influence mortalities is essential for identifying vulnerable populations, informing targeted management, and refining conservation strategies. While natural mortality may be perceived as a negligible threat in large populations such as that of Hwange National Park, estimated at approximately 83,000 elephants, this view underestimates the vulnerability of smaller or isolated populations. Moreover, mass mortality events, such as the unexplained deaths of over 350 elephants in Botswana in 2020 ^30^, highlight the importance of early detection and predictive modeling to avert catastrophic losses, even in seemingly stable populations.

This study investigates the spatial and environmental determinants of natural elephant mortality in Hwange National Park, Zimbabwe. By integrating carcass records with high-resolution environmental and climatic variables, we employ ensemble machine learning models to predict mortality hotspots across the landscape. This approach represents a novel application of ensemble SDMs to natural mortality analysis and seeks to identify the key environmental correlates that shape elephant vulnerability. Our findings aim to contribute to the development of early warning systems and adaptive conservation planning strategies under the pressures of accelerating environmental change.

## Materials and method

### Study site

**Fig. 1:**
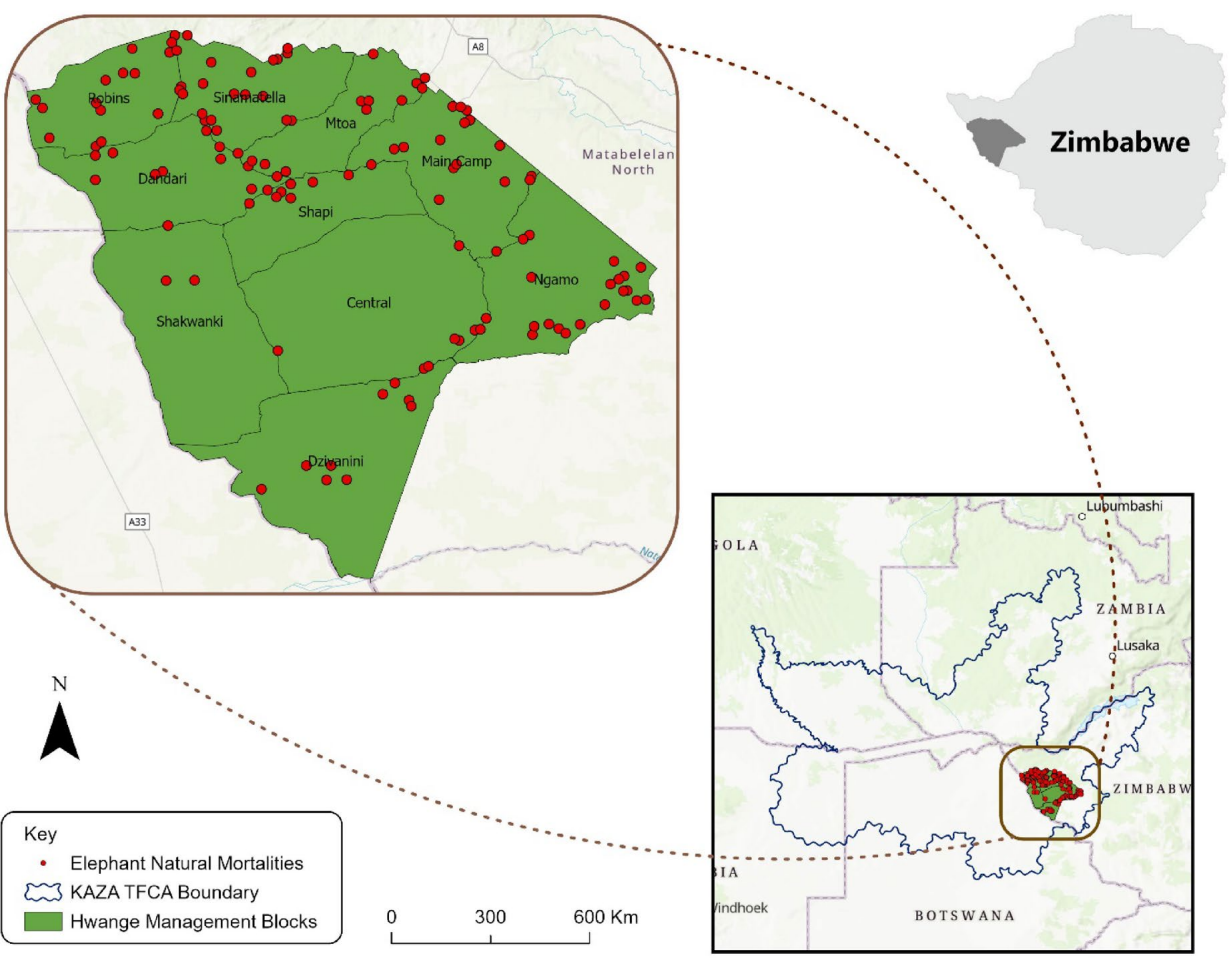
Hwange National Park, its administrative zones and surrounding areas in south-western Zimbabwe.

The study was conducted in Hwange National Park (14.651 km^2^), wildlife management area in southwestern Zimbabwe^31^ (Fig. 1). This park is part of the Kavango-Zambezi Transfontier Conservation Area (KAZA) which straddles across 5 nations (~ Zambia, Botswana, Angola, Botswana and Namibia). The southern border of the park is bordered by Botswana and the northern part is bordered by rural community land in Matebeleland North province of Zimbabwe.

The soils in Hwange National Park are predominantly Kalahari sands with alluvial soils found along rivers ^32^. The area receives seasonal rainfall which varies spatially and temporarily with an average annual rainfall amount of 600 mm ^33^. The summer rains received between November and April are commonly associated with mid-season dry spells. Some dry days can extend for a long period resulting in droughts, such as the decadal droughts in 1992 and 2002. The relatively moderate rainfall amount supplies water into pools which act as natural water sources for wildlife. To supplement water supplies, some pools are supplied by numerous solar powered boreholes across which pump water throughout the year. Borehole supplied waterpoints act as an important water source during the dry season months attracting large numbers of various wildlife species such as elephants, *Tragelaphus strepsiceros* (kudu), A*epyceros melampus* (impala), *Taurotragus oryx* (eland), P*anthera leo* (lion) and S*yncerus caffer* (buffalo). The average temperature varies widely as a factor of variation in topography and seasonality with an average temperature of 25 °C. High temperatures are commonly experienced during the dry summer season begetting thermal stressing conditions to some plants and animals. The climatic conditions support mostly the *Colophospermum mopane*, *Guibortiacoleosperm, Baikaiea plurijuga, Baphian assaiensis and Bauhinia petersiana* woodlands which dominate the landscape ^34^.

### Natural elephant mortality data

We used natural elephant mortality data collected through systematic, ground-based law enforcement patrols conducted across Hwange National Park between 2020 and 2022. These patrols, coordinated by the Zimbabwe Parks and Wildlife Management Authority (ZPWMA), are typically distributed evenly throughout the park to ensure consistent spatial coverage, rather than being concentrated in specific landscape features or vegetation types. While the intention is to patrol all areas of the park, limitations in resources and tracking technology occasionally hinder full coverage and make it difficult to precisely assess the extent of patrol effort. Based on the data available, likely a conservative estimate, average spatial coverage during the study period was approximately 62%, with effort generally well dispersed across the park.

In contrast to other protected areas such as the Chewore Safari Area, where Kuiper et al. ^35^ documented significant spatial biases due to uneven patrol distribution, Hwange benefits from relatively strong institutional support and external funding from multiple donor organizations. These factors have contributed to more consistent law enforcement and surveillance efforts, helping to reduce sampling bias and enhance data reliability.

**Fig. 2:**
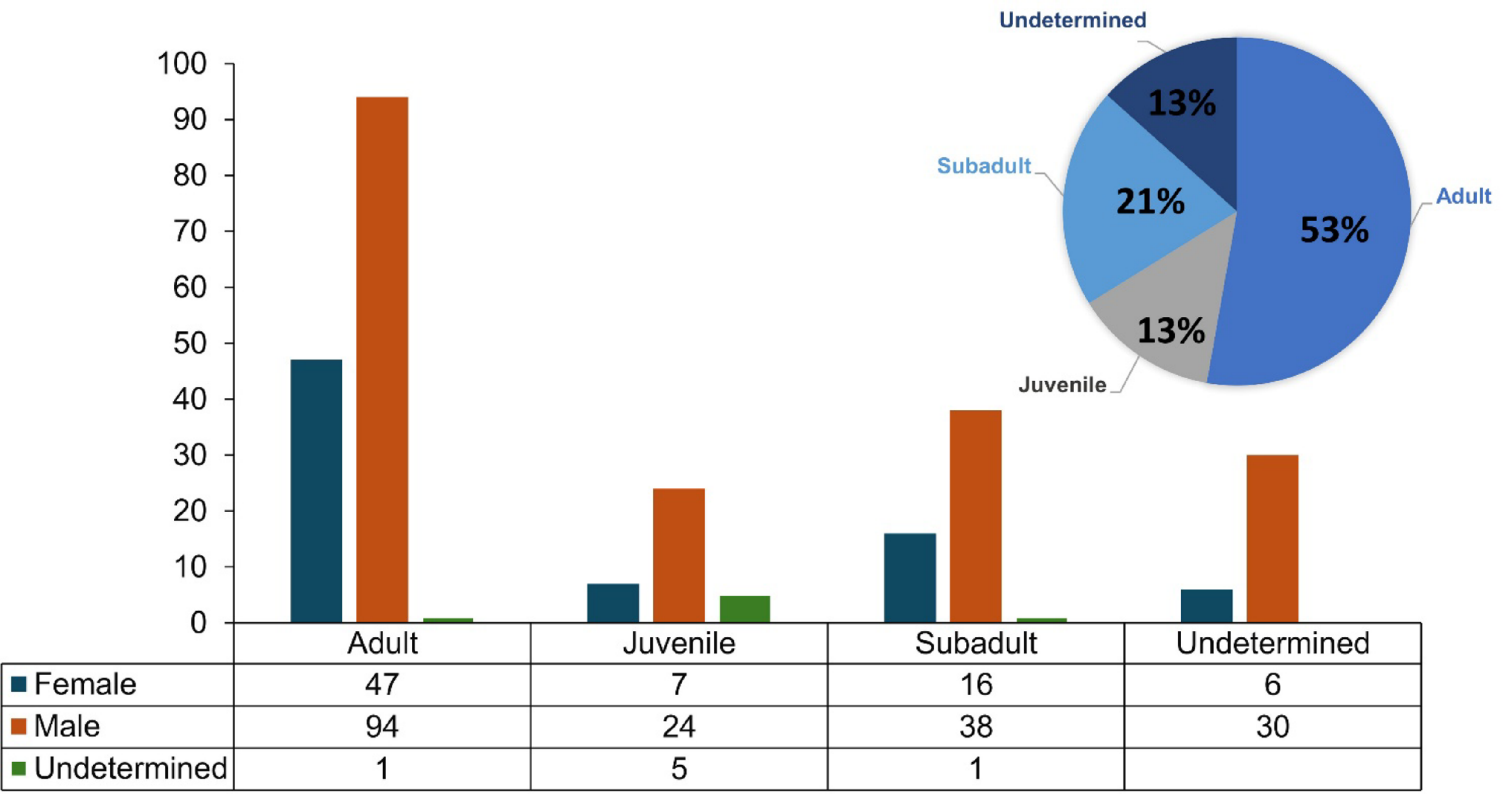
Age and sex distribution of natural elephant mortalities in Hwange National Park.

We obtained mortality data from the national elephant mortality database, which includes records of deaths from various causes, including poaching, management interventions, and poisoning. Mortality data were already classified as natural when carcasses were discovered with intact tusks and showed no evidence of trauma, poisoning, or chemical exposure at the time of discovery. We present the age class and sex distributions of the deceased elephants in Fig. 2. Of the 269 natural mortality records available for the study period, we only used 220 records that had valid geographic coordinates.

To address potential spatial autocorrelation among presence points, as recommended by ^36^, we applied a spatial thinning procedure with a minimum interpoint distance of 1.5 km. This threshold was based on daily elephant movement ranges reported by Foley ^37^ in a protected savanna landscape comparable to our study area. The thinning process reduced the number of training points from 220 to 125. To generate absence data for natural elephant mortalities, we calibrated the modeling framework to generate 1000 pseudo-random absence points distributed across the entire extent of Hwange National Park. This approach ensured that the background data spatially matched the full prediction area, thereby minimizing sampling bias and improving the ecological representativeness of the absence data used in model calibration following best practices outlined by Barbet-Massin et al. ^38^.

### Environmental variables

We used ten environmental variables to model the potential geographic distribution of natural elephant mortalities: annual average temperature (AAT), annual average precipitation (AAP), climate water deficit (CWD), elevation, percentage tree cover (PTC), distance from water sources (DFW), maximum temperature of the warmest month (MTWM), Normalized Difference Vegetation Index (NDVI), and elephant density. We selected these variables based on their ecological relevance to elephant distribution and mortality, as supported by previous studies ^23,32,34,39^.

We obtained AAT and MTWM from the World Climate Database, and sourced AAP from the Climate Hazards Center InfraRed Precipitation with Station data (CHIRPS) ^40^. We used MODIS products to extract NDVI (MOD13Q1), as a proxy for forage quality, and PTC (MOD44B), as an indicator of forage quantity ^41^. Although these datasets varied in temporal and spatial resolution, we standardized them by averaging over the study period. We downloaded AAT, MTWM, and AAP as long-term means, while we processed the 8-day NDVI composites into a single average value covering the entire study period.

To represent surface water availability, we used DFW. We compiled vector layers of permanent and seasonal water sources from Diva GIS, the ZimParks spatial database, and additional features digitized using expert knowledge from ZPWMA patrol rangers with over a decade of experience in the field. Before generating the Euclidean distance matrix, we projected all water layers to WGS 84 / UTM Zone 35S in ArcMap 10.2 ^42^.

We incorporated elevation as a proxy for terrain influence, following Kyale ^43^, and sourced a 30 m resolution Digital Elevation Model (DEM) from the USGS. We also included CWD to account for drought stress, which is known to affect elephant survival ^27^. CWD captures the extent to which atmospheric water demand exceeds soil moisture availability, influencing vegetation productivity and surface water persistence. We obtained annual CWD data from TerraClimate using Google Earth Engine ^44^ and calculated the mean CWD across the study period. To assess how elephant distribution may relate to mortality patterns, we incorporated elephant density derived from 2015 aerial survey data ^45^.

**Fig. 3:**
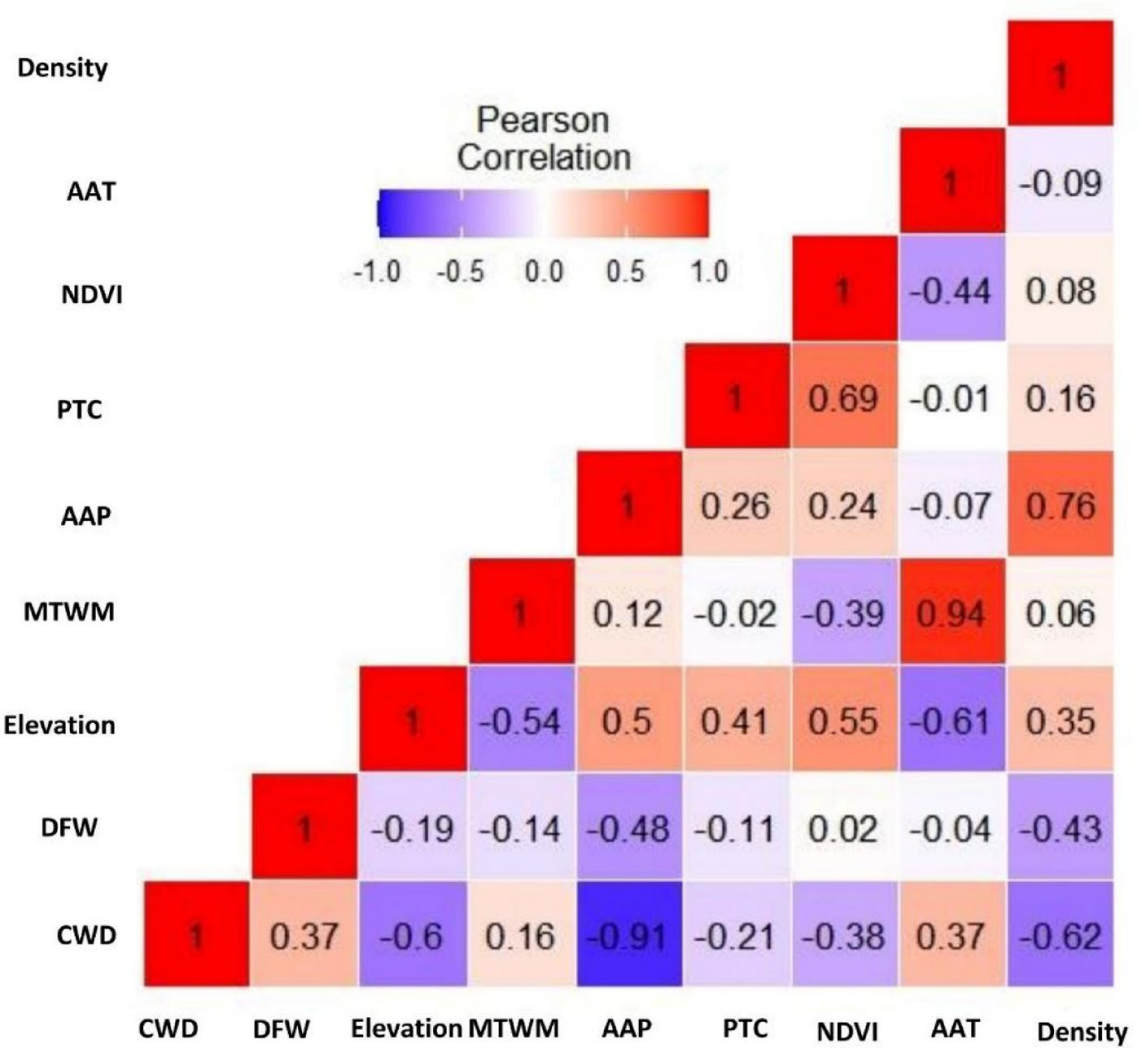
Pairwise correlation matrix between the predictor variables.

Before modeling, we resampled all environmental layers to a common 30 m spatial resolution using the nearest neighbour technique. We then assessed multicollinearity among predictors using pairwise Pearson correlations in R ^46^, applying a threshold of |r|> 0.7 to retain a parsimonious model ^47^ (See Fig. 3). Based on this, we excluded AAP due to its strong negative correlation with CWD (r = − 0.91), and we removed AAT due to its high collinearity with MTWM. While NDVI showed moderate correlation with PTC (r = 0.69), and elephant density with AAP (r = 0.76), we retained both due to their distinct ecological roles in shaping elephant habitat use and mortality.

### Modeling approach

We used an ensemble of four ML species distribution modeling techniques, RF, GBM, MaxEnt, and XGBoost to model the spatial distribution of natural elephant mortalities. We selected these algorithms due to their demonstrated performance in ecological modeling and their extensive application in elephant ecology ^42–47^.

We implemented the models using the BIOMOD2 package in R ^48^. For each model run, we used bootstrapping with 10 repetitions, splitting the presence–absence data into 80% for model calibration and 20% for validation ^49^. We employed ensemble modeling to minimize biases and limitations associated with single-model predictions, as recommended by Buisson et al. ^22^ and Grenouillet et al. ^21^. BIOMOD2 was configured to generate spatial probability maps ranging from 0 to 1, where values near 0 indicate low predicted prevalence of natural elephant mortalities and values near 1 indicate high prevalence ^50^.

We calculated variable importance scores to determine the relative contribution of each environmental predictor to the modeled mortality distribution. We followed the method described by Gregorutti et al. ^51^, which compares predictions from the full ensemble model with predictions from models where one variable is removed. A high correlation between these predictions indicates low variable importance, while a low correlation suggests that the removed variable strongly influenced the model output ^48,52^.

### Model evaluation and comparison

We assessed model performance using two widely accepted metrics: the True Skill Statistic (TSS), which balances sensitivity and specificity to measure overall classification accuracy, and the Area Under the Receiver Operating Characteristic Curve (ROC), which quantifies a model’s ability to discriminate between presence and absence across all probability thresholds. We classified model performance following the thresholds established by Phillips et al. ^53^ and Phillips and Dudík ^54^ . Specifically, we considered models with performance values ≤ 0.5 as poor, values between 0.75 and 0.90 as good, and values > 0.90 as excellent.

TSS values range from − 1 to + 1, where + 1 indicates perfect agreement between predicted and observed values, and values of zero or below indicate a model performing no better than random ^55^. To ensure reliability of the final ensemble, we configured the BIOMOD2 framework to include only models with ROC scores of at least 0.75. We applied the maximum sensitivity–specificity threshold to convert continuous probability outputs into binary mortality prevalence predictions, balancing omission and commission errors for optimal model performance.

To compare the predictive accuracy of individual algorithms, we summarized TSS and ROC scores in tabular format and visualized their variation across models and repetitions. This allowed us to assess both the consistency and robustness of each model’s contribution to the ensemble.

## Results

### Model performance

**Fig. 4:**
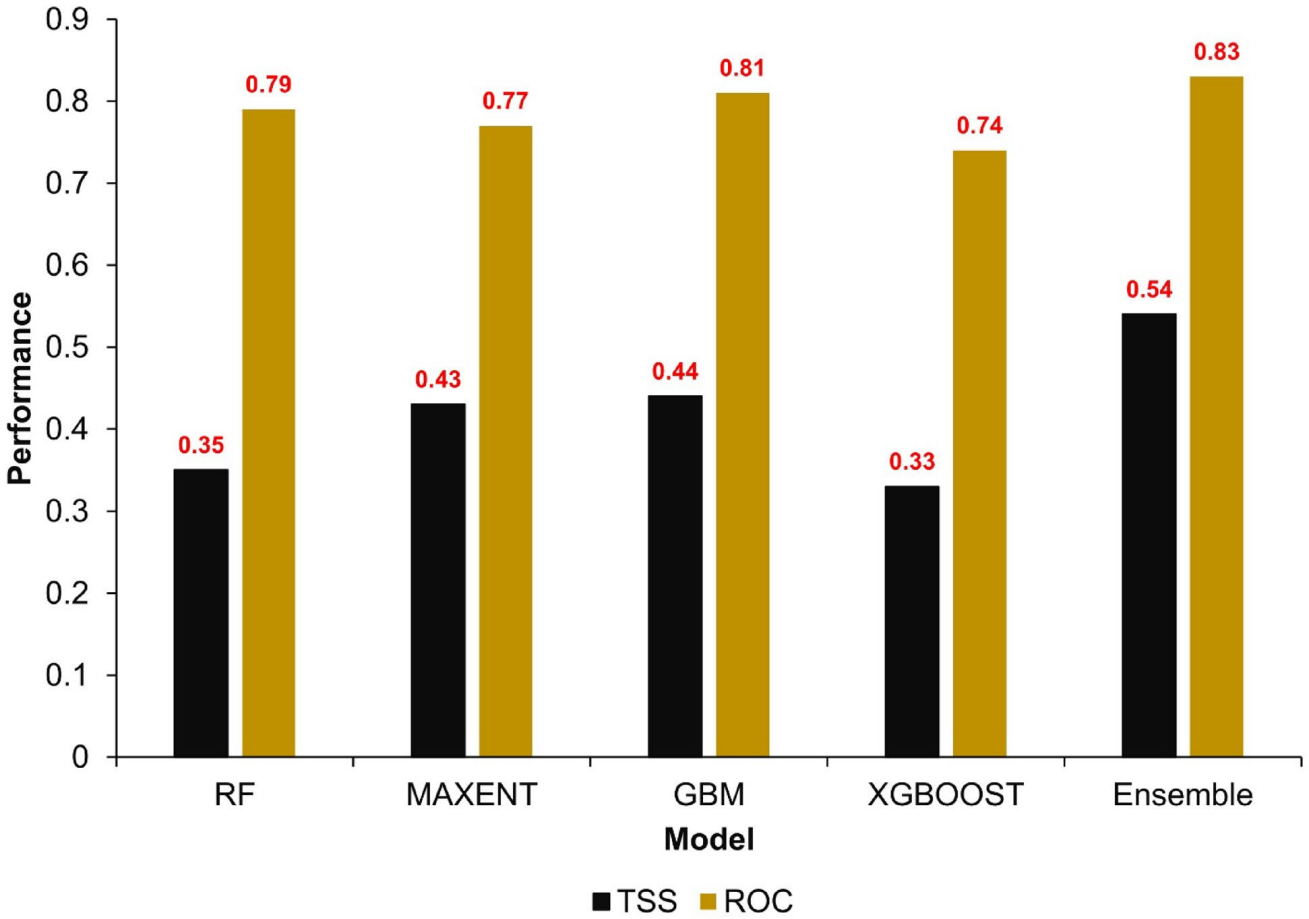
Performance comparison of individual machine learning models (RF, MaxEnt, GBM, XGBoost) and their ensemble model.

We found that the ensemble model outperformed all individual models, demonstrating superior predictive accuracy with a TSS of 0.54 and a ROC of 0.83 (Fig. 4). In contrast, the individual models showed variable performance. GBM performed relatively well, achieving a TSS of 0.44 and a ROC of 0.81, while RF yielded slightly lower values (TSS = 0.35; ROC = 0.79). MaxEnt exhibited comparable predictive power to RF, with a TSS of 0.43 and a ROC of 0.77. XGBoost recorded the weakest performance, with a TSS of 0.33 and a ROC of 0.74. These results underscore the enhanced reliability of the ensemble approach in capturing the spatial distribution of natural elephant mortalities, outperforming each constituent model across all evaluation metrics.

### Spatial patterns of natural mortalities

**Fig. 5:**
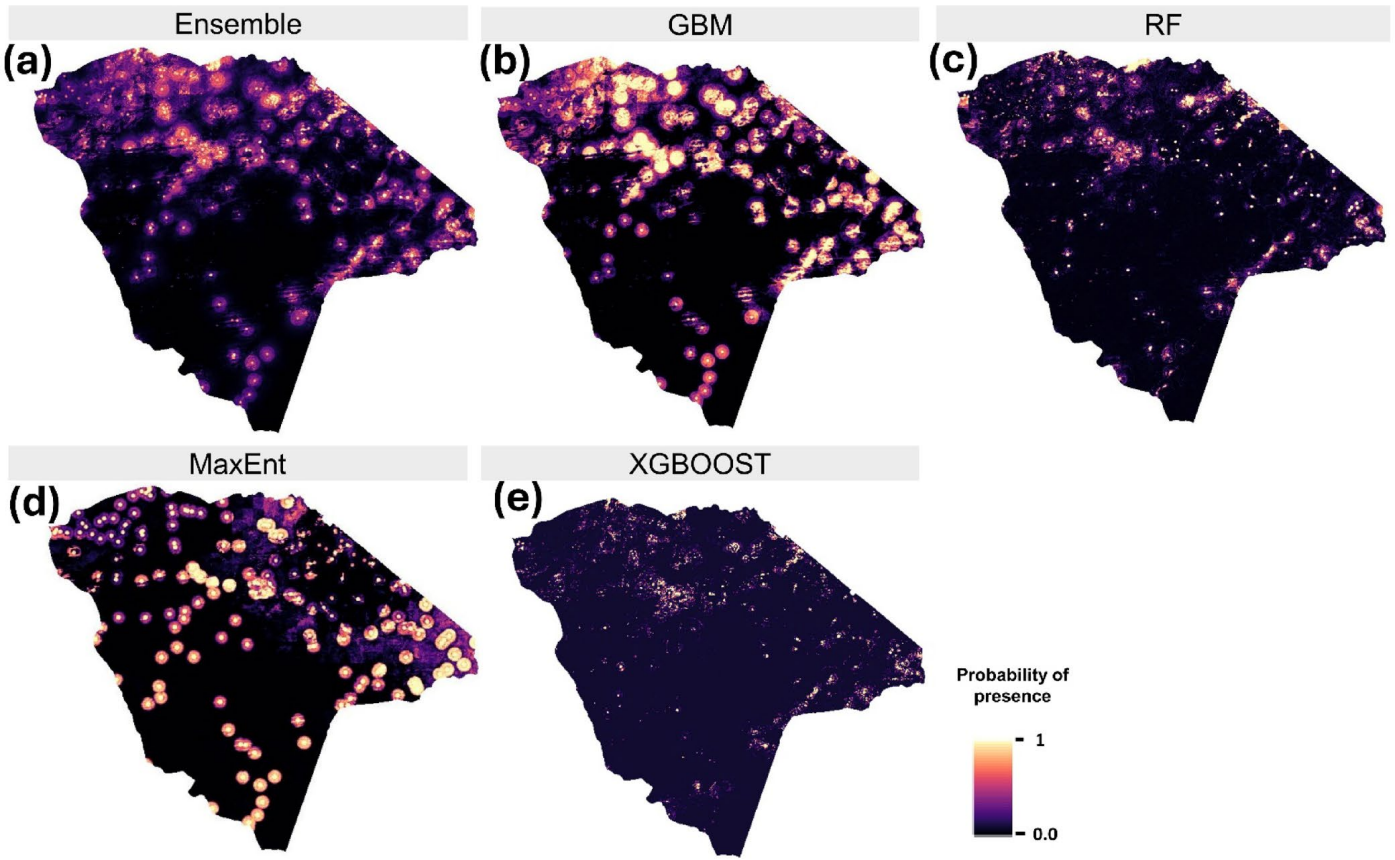
Predicted distribution of natural elephant mortalities in Hwange National Park using (**a**) the ensemble model and individual models: (**b**) GBM, (**c**) RF, (**d**) MaxEnt, and (**e**) XGBoost. Values range from 0 (low mortality potential) to 1 (high mortality potential).

The ensemble model and its constituent machine learning algorithms consistently predicted that natural elephant mortalities are spatially clustered in specific areas spread out across Hwange National Park (Fig. 5a–e). All models revealed similar distribution patterns, with high-prevalence clusters concentrated primarily in the northern (Sinamatella, Robins, Mtoa, Shapi, Main Camp, and Dandari) and eastern (Ngamo) regions of the park. While some models, such as GBM (Fig. 5b) and MaxEnt (Fig. 5d) predicted higher probabilities of presence compared to others like RF (Fig. 5c) and XGBoost (Fig. 5e), the overall spatial pattern remained consistent across models.

### Variable importance

**Fig. 6:**
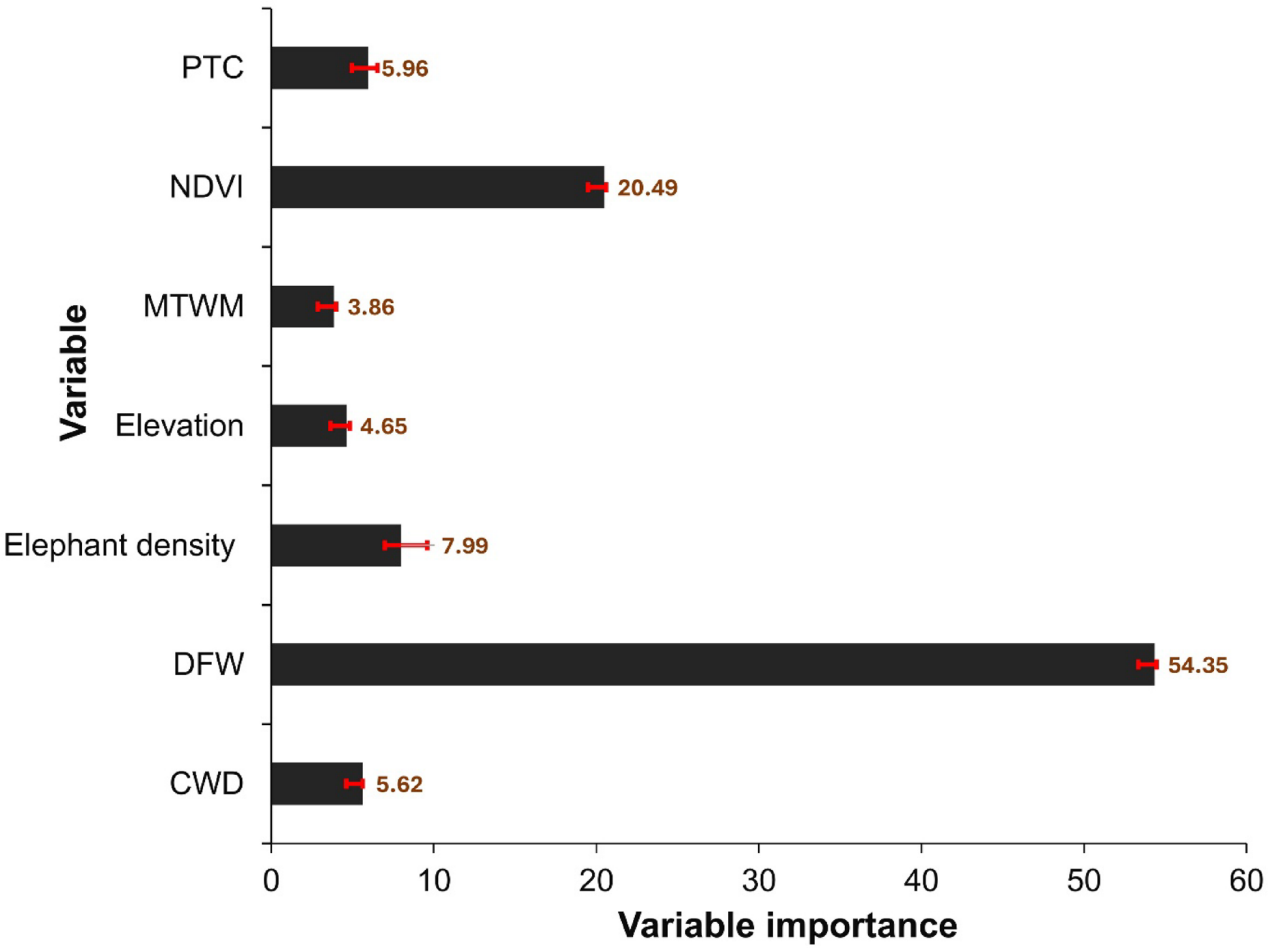
Variable importance in predicting natural elephant mortalities in Hwange National Park, based on 10 model runs using PTC, NDVI, MTWM, DFW, and CWD.

We found that DFW, PTC, NDVI, elephant density and CWD were the key predictors of natural elephant mortalities, each contributing more than 5% to the overall model performance (Fig. 6). Among these, DFW emerged as the single most important variable, indicating that proximity to water sources plays a central role in shaping mortality prevalence.

In contrast, MTWM and elevation contributed less than 4% to model performance, indicating a relatively minor role in explaining spatial variation in natural mortalities within this landscape.

### Variable responses

**Fig. 7:**
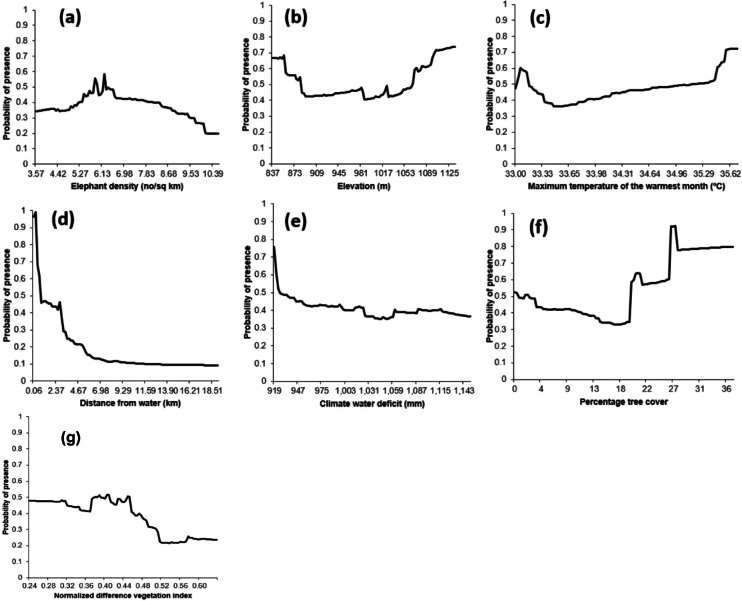
Ensemble model response curves showing the effect of (**a**) elephant density, (**b**) elevation, (**c**) MTWM, (**d**) DFW, (**e**) CWD, (**f**) PTC, and (**g**) NDVI on the probability of natural elephant mortality.

We observed that natural elephant mortality prevalence responded different to our predictive variables: elephant density (Fig. 7a), elevation (Fig. 7b), maximum temperature of the warmest month (Fig. 7c), distance from water (Fig. 7d), climate water deficit (Fig. 7e), percentage tree cover (Fig. 7f), and NDVI (Fig. 7g). The highest prevalence of natural elephant mortalities was in areas located within 6 km meters of water sources, with mortality rates declining progressively with increasing distance from water (Fig. 7d). This shows the critical role of surface water availability in shaping mortalities, particularly in semi-arid savanna systems where elephants frequently aggregate near waterholes.

Vegetation-related variables, PTC and NDVI showed contrasting but ecologically consistent patterns. Mortality prevalence was highest in areas characterized by low NDVI values (< 0.5) (Fig. 7g) and relatively sparse tree cover (< 20%) (Fig. 7f), conditions typical of open woodland habitats. Such areas typically offer limited shade and forage quality, compounding physiological stress and reducing access to key resources.

CWD exhibited a non-linear effect, with higher mortality prevalence associated with low to moderate values (between 919 mm and 1000 mm) (Fig. 7e). Elevation also influenced mortality prevalence. Areas above 960 m recorded greater prevalence compared to moderate and lower elevations (< 1000 m) (see Fig. 7b). This highlights the microclimatic or vegetation differences that intensify stress or limit forage availability at higher altitudes.

We also found that elephant density played a role, with higher mortality prevalence occurring in areas with low to moderate population densities (4–7 elephants/km^2^) (See Fig. 7a). This reflects localized crowding effects at critical resource sites or weakened individuals moving away from core high-density zones.

Finally, elevated maximum temperatures during the warmest month (MTWM > 35 °C) were associated with increased mortality prevalence, underscoring the potential role of thermal stress in driving natural deaths under extreme climatic conditions (Fig. 7c).

## Discussion

African elephants (*Loxodonta africana*) are currently at risk from poaching, human-elephant conflict, habitat loss and climate variability. These risks will likely intensify or increase in the future under global environmental change. In this study, we sought to identify the spatial determinants of elephant natural mortalities with the use of ensemble modeling.

### Drivers of natural elephant mortalities

We found that elephant deaths were concentrated near water points (DFW < 6 km), in areas with high tree cover (PTC > 20%) and moderate drought exposure (CWD > 919 mm), suggesting that proximity to water may interact with ecological and physiological stressors to drive natural mortalities. One plausible mechanism is the congregation of elephants around shrinking and stagnant water sources during dry periods, which can facilitate the transmission of pathogens such as those responsible for cyanobacterial toxicosis and septicaemia, both previously implicated in mass elephant die-offs ^11,29,53,56^. Warm, nutrient-rich and stagnant water bodies, common during droughts create ideal conditions for cyanobacterial blooms ^11^, increasing the risk of intoxication, especially when elephants are forced to drink from limited sources.

Additionally, thermal stress may exacerbate elephant vulnerability. Thermal stress is known to impair immune function in elephants and has been linked to increased susceptibility to infectious diseases in both African and Asian elephants ^57^. Another possibility is that aged or compromised individuals are less able to forage or travel long distances from water sources during harsh conditions, effectively becoming trapped in high-prevalence zones. Also, lion predation could explain juvenile mortalities though constituting 13% of the total mortalities. According to Loveridge et al. ^58^, lion predation on elephants is common in Hwange during dry years and constitute a significant portion of the biomass consumed by lions ^58^. This likely occurs close to water sources where lions strategically ambush elephant heads when they are drinking water.

CWD, a proxy to measure droughts, explained the distribution of natural elephant mortalities. Due to the little or no precipitation especially in the dry season, CWD fluctuates. However, when coupled with droughts, high values can span for more than 100 days. Surface water availability is key to elephant physiology and its absence means more energy is spent by elephants to traverse to areas with surface water. However, this could be difficult for female elephants that can only move as far as their calves ^58,59^. This increases the risk of mortality for females and juvenile elephants (see Fig. 2) as observed by Corfield ^59^ and Dudley et al. ^27^. The effect of droughts on females, calves and juvenile elephant mortalities has been observed in several African and Asian landscapes. For instance in Tsavo Protected Area, ^49^ observed that elephant mortalities were observed after prolonged periods without rainfall (> 105 days). Dudley et al. ^27^ also observed that elephant mortalities in Hwange were more influenced by the number of consecutive dry days and consecutive wet days than the total precipitation received in Hwange.

We found that natural elephant mortalities were more frequent in areas with a NDVI below 0.5, which typically reflects sparse or stressed vegetation cover. This pattern likely reflects the energetic stress elephants face in low-productivity environments, particularly during dry seasons when food availability is limited ^27^. Elephants require large quantities of forage, and areas with low NDVI may offer insufficient vegetation to meet their dietary needs, increasing vulnerability to starvation, dehydration, and disease. Moreover, these areas may also support fewer shade-providing trees, compounding physiological stress during extreme heat or drought periods^59^.

We found that elevation was an important variable in explaining the distribution of natural elephant mortalities. Natural mortalities were concentrated in water points in high elevation areas (> 1000 m). When elephants get to water points, Conybeare and Haynes ^60^ and Ramey et al. ^61^ observed that elephants dug up water around water points (especially seasonal water points) using their trunks. Juvenile elephants that fail to dig up water can succumb to heat stress and die. Also, elephants have been observed to struggle with steep and mountainous areas due to the high energy cost required ^62^. According to Wall et al. ^62^ it is estimated that an average elephant needs 2500% more energy to move up a slope than on level terrain. Our observation on how elevation (> 960 m) explains elephant natural mortalities is in sync with observations made by Roever et al. ^63^ in Botswana. Roever et al. ^63^ observed that elephants mostly die in high steep and mountainous areas, typical of high elevated areas.

While previous studies have reported the effect of drought and septicaemia ^11,64^, identifying the spatial determinants and areas of natural mortality concentrations is novel. Previous studies, such as Roever et al. ^63^, evaluated the drivers of elephant mortalities in a human-dominated area in northern Botswana using logistic regression. Their analysis incorporated mortality locations from aerial surveys and covariates such as slope, PTC, distance to humans and distance to roads. They found that proximity to humans was the primary predictor of elephant mortalities, whereas our study identified distance from water as the top predictor. Unlike their research, we conducted our study within a protected area, focusing exclusively on natural causes of mortality and utilizing advanced machine learning algorithms. Furthermore, while Roever et al. ^63^ observed that elephants tend to die away from water sources, our findings show that most mortalities occur near water. These differences likely stem from variations in study sites and the nature of the mortality data used. The data in their study may have included human-related deaths such as those caused by poaching, poisoning, sport hunting, or human-wildlife conflict, which could explain the dominance of human proximity in their results. In contrast, we present the first drivers of natural elephant mortalities. The differences in findings of study from that of Roever et al. ^63^ suggests that observations for elephant mortalities in human invaded spaces may not be transferable to protected areas, particularly when focusing on natural mortalities.

### Model performance

We present statistically robust results owing to the use of an ensemble modeling built using ML algorithms. Overall, ensemble models performed better than individual models. An ensemble model typically has higher predictive accuracy and can deduce patterns from data ^63,64^. Additionally, ensemble models can be tuned with small or large datasets and will not overfit ^19,65^. ML ensembles have been used before to explain elephant distribution ^23^, human-elephant conflict ^25^, elephant poaching risk areas ^24^ and effect of climate on elephants ^26^ with high predictive accuracy. We use ML ensembles for the first time to tease apart the key determinants explaining elephant natural mortalities.

### Study limitations

We provide valuable insights into the environmental factors associated with the distribution of natural elephant mortalities in Hwange National Park. While this is among the first attempts to model and explain spatial patterns of natural elephant deaths, a key limitation was the inability to incorporate disease-related variables. This is largely due to the underdevelopment of wildlife veterinary services across much of sub-Saharan Africa, with Zimbabwe being no exception. These services are often costly and remain underfunded in most state-managed protected areas. Although rangers receive basic training in identifying pests and diseases, they are not adequately equipped to respond to outbreaks ^66^. Consequently, interventions typically occur only after substantial die-offs such as anthrax outbreaks in Botswana’s elephant populations ^30,67^. Future studies should therefore aim to include disease occurrence as a covariate when modeling natural mortalities.

Despite these limitations, we offer practical guidance for conservation and management. By identifying mortality hotspots, particularly those near water sources and areas with high climatic stress, our results can help managers prioritize surveillance and response efforts in high-prevalence zones, especially for small or reintroduced elephant populations. We also provide a framework for informing reintroduction strategies whereby high-prevalence areas can be flagged for avoidance of introductions or targeted for enhanced monitoring and veterinary preparedness, while low-prevalence areas may serve as more suitable release sites. Integrating spatial mortality prevalence into reintroduction planning could reduce post-release losses, develop early warning systems and improve long-term success. As climate and disease pressures intensify, this kind of adaptive, risk-informed approach will be increasingly critical.

## Conclusion

We aimed to identify the drivers and spatial distribution of elephant mortalities in Hwange National Park from 2020 to 2022. We found that DFW, PTC, and CWD were significant contributors to the distribution of natural elephant deaths. We show that elephant mortalities are likely to rise under global climate change, primarily due to worsening drought conditions and increased susceptibility to diseases like septicaemia.

We suggest that to ensure the effective conservation of elephants, it is crucial to address both natural and anthropogenic threats to their survival. Focusing solely on human-related prevalence may overlook significant natural challenges that can threaten elephant populations. Therefore, a balanced conservation strategy should be adopted, recognizing that natural factors, such as climate-induced stressors and diseases, are equally critical to the long-term viability of elephant populations.

## Data Availability

Natural elephant mortality data and covariates used in this study are available on the following repository: 10.5281/zenodo.13715371.

## References

[1] Bennett, E. L. Legal ivory trade in a corrupt world and its impact on African elephant populations. *Conserv. Biol.* **29**, 54–60 (2015). 25103555 10.1111/cobi.12377

[2] Blanc, J. J. African elephant status report 2007: An update from the African elephant database, *Iucn*, 2007.

[3] Blanc, J. J. et al. Changes in elephant numbers in major savanna populations in eastern and southern Africa. *Pachyderm* **38**, 19–28 (2005).

[4] Gobush, K. S., Mutayoba, B. M. & Wasser, S. K. Long-term impacts of poaching on relatedness, stress physiology, and reproductive output of adult female African elephants. *Conserv. Biol.* **22**, 1590–1599 (2008). 18759771 10.1111/j.1523-1739.2008.01035.x

[5] Milliken, T., Underwood, F., Burn, R. & Sangalakula, L. Addendum to the Elephant Trade Information System (ETIS) and the Illicit Trade in Ivory. In *A Report to the 17th Meeting of the Conference of the Parties to CITES*, TRAFFIC. (2016).

[6] Milner-Gulland, E. J. &. Beddington, J. R. The exploitation of elephants for the ivory trade: an historical perspective. In *Proceedings of the Royal Society of London. Series B: Biological Sciences*. pp. 29–37 (1993).

[7] Chamaille-James, S., Matsika, R., Matsvimbo, F. & Madzikanda, H. Having your water and drinking it too: Resource limitation modifies density regulation. *J. Appl. Ecol.* **27**, 1–4 (2008). 10.1111/j.1365-2656.2007.01306.x 17986250

[8] Bohrer, G., Beck, P. S., Ngene, S. M., Skidmore, A. K. & Douglas-Hamilton, I. Elephant movement closely tracks precipitation-driven vegetation dynamics in a Kenyan forest-savanna landscape. *Mov. Ecol.* **2**(2), 2 (2014). 25520813 10.1186/2051-3933-2-2 PMC4267703

[9] Shrader, A. M., Pimm, S. L. & Aarde, R. J. Elephant survival, rainfall and the confounding effects of water provision and fences. *Biodivers. Conserv.* **19**, 2235–2245 (2010).

[10] Okello, M. M. et al. Population density of elephants and other key large herbivores in the Amboseli ecosystem of Kenya in relation to droughts. *J. Arid Environ.* **135**, 64–74 (2016).

[11] Foggin, C. M. et al. Pasteurella sp. associated with fatal septicaemia in six African elephants. *Nat. Commun.* **14**(1), 6398. 10.1038/s41467-023-41987-z (2023). 37880229 10.1038/s41467-023-41987-z PMC10600241

[12] Chamaillé-Jammes, S., Fritz, H. & Murindagomo, F. Climate-driven fluctuations in surface-water availability and the buffering role of artificial pumping in an African savanna: Potential implication for herbivore dynamics. *Austral Ecol.* **32**, 740–748 (2007).

[13] Clegg B. Habitat and diet selection by the African elephant at the landscape level: A functional integration of multi-scale foraging process.(2008).

[14] Mpakairi, K. S. et al. Missing in action: Species competition is a neglected predictor variable in species distribution modelling. *PLoS ONE* **12**(7), e0181088. 10.1371/journal.pone.0181088 (2017). 28708854 10.1371/journal.pone.0181088 PMC5510852

[15] Gara, T. W. et al. Understanding the effect of landscape fragmentation and vegetation productivity on elephant habitat utilization in Amboseli ecosystem, Kenya. *Afr. J. Ecol.* **55**(3), 259–269 (2017).

[16] Ihwagi, F. W. et al. Poaching lowers elephant path tortuosity: Implications for conservation. *J. Wildl. Manag.* **83**, 1022–1031 (2019).

[17] Murwira, A. & Skidmore, A. K. The response of elephants to the spatial heterogeneity of vegetation in a Southern African agricultural landscape. *Landsc. Ecol.* **20**, 217–234 (2005).

[18] De Meulenaer, T. Update on the implementation of the MIKE programme in Africa/Mise à jour sur la mise en oeuvre du Programme de MIKE en Afrique. *Pachyderm* **48**, 78–81 (2010).

[19] Pearson, R. G., Raxworthy, C. J., Nakamura, M. & Peterson, A. T. Predicting species distributions from small numbers of occurrence records: A test case using cryptic geckos in Madagascar. *J. Biogeogr.* **34**, 102–117 (2007).

[20] Marmion, M., Parviainen, M., Luoto, M., Heikkinen, R. K. & Thuiller, W. Evaluation of consensus methods in predictive species distribution modelling. *Divers. Distrib.* **15**(1), 59–69 (2009).

[21] Grenouillet, G., Buisson, L., Casajus, N. & Lek, S. Ensemble modelling of species distribution: The effects of geographical and environmental ranges. *Ecography* **34**, 9–17 (2011).

[22] Buisson, L., Thuiller, W., Casajus, N., Lek, S. & Grenouillet, G. Uncertainty in ensemble forecasting of species distribution. *Glob. Chang. Biol.* **16**, 1145–1157 (2010).

[23] Ndaimani, H., Murwira, A., Masocha, M. & Zengeya, F. M. Elephant (Loxodonta africana) GPS collar data show multiple peaks of occurrence farther from water sources. *Cogent Environ. Sci.* **3**(1), 1420364 (2017).

[24] Sibanda, M. et al. Understanding the spatial distribution of elephant (*Loxodonta africana*) poaching incidences in the mid-Zambezi Valley, Zimbabwe using geographic information systems and remote sensing. *Geocarto Int.* **31**, 1006–1018 (2016).

[25] Naha, D., Sathyakumar, S., Dash, S., Chettri, A. & Rawat, G. Assessment and prediction of spatial patterns of human-elephant conflicts in changing land cover scenarios of a human-dominated landscape in North Bengal. *PLoS ONE* **14**, e0210580 (2019). 10.1371/journal.pone.0210580 PMC6358066 30707690

[26] Mpakairi, K. S., Ndaimani, H., Tagwireyi, P., Zvidzai, M. & Madiri, T. H. Futuristic climate change scenario predicts a shrinking habitat for the African elephant (*Loxodonta**africana*): Evidence from Hwange National Park, Zimbabwe. *Eur. J. Wildl. Res.* **66**(1), 1 (2020).

[27] Dudley, J. P., Gibson, D. S. C., Haynes, G. & Klimowicz, J. Drought mortality of bush elephants in Hwange National Park, Zimbabwe. *Afr. J. Ecol.* **39**(2), 187–194 (2001).

[28] Woolley, L.-A., Mackey, R. L., Page, B. R. & Slotow, R. Modelling the effect of age-specific mortality on elephant *Loxodonta africana *populations: Can natural mortality provide regulation?. *Oryx* **42**, 49–57 (2008).

[29] Benza, B. Botswana says toxins in water killed hundreds of elephants. Reuters (2020).

[30] Azeem, S., Bengis, R., Van, A. R. & Bastos, A. D. S. Mass die-off of African elephants in Botswana : Pathogen, poison or a perfect storm?. *Afr. J. Wildl. Res.* **50**(1), 149–156. 10.3957/056.050.0149 (2020).

[31] Kavhu, B. & Ndaimani, H. Analysing factors influencing fire frequency in Hwange National Park. *S. Afr. Geogr. J.* **104**(2), 177–192. 10.1080/03736245.2021.1941219 (2022).

[32] Childes, S. L. & Walker, B. H. Ecology and dynamics of the woody vegetation on the Kalahari sands in Hwange National Park, Zimbabwe. *Vegetatio* **72**(2), 111–128 (1987).

[33] Hubbard, P. & Haynes, G. Mtoa Ruins, Hwange National Park, Zimbabwe. *Zimbabwean Prehistory* **30**(2), 25–33 (2012).

[34] Rogers, C. M. A woody vegetation survey of Hwange National Park. Accessed 07 July 2025. https://www.cabidigitallibrary.org/doi/full/10.5555/20013056811 (1993)

[35] Kuiper, T., Kavhu, B., Ngwenya, N. A., Mandisodza-Chikerema, R. & Milner-Gulland, E. J. Rangers and modellers collaborate to build and evaluate spatial models of African elephant poaching. *Biol. Cons.* **243**, 108486 (2020).

[36] Kramer-Schadt, S. et al. The importance of correcting for sampling bias in MaxEnt species distribution models. *Divers. Distrib.* **19**(11), 1366–1379. 10.1111/ddi.12096 (2013).

[37] Foley, L. S. The influence of environmental factors and human activity on elephant distribution in Tarangire National Park, Tanzania. ITC (2002).

[38] Barbet-Massin, M., Jiguet, F., Albert, C. H. & Thuiller, W. Selecting pseudo-absences for species distribution models: How, where and how many?. *Methods Ecol. Evol.* **3**(2), 327–338. 10.1111/j.2041-210X.2011.00172.x (2012).

[39] Valeix, M. et al. Vegetation structure and ungulate abundance over a period of increasing elephant abundance in Hwange National Park, Zimbabwe. *J. Trop. Ecol.* **23**(1), 87–93 (2007).

[40] Funk, C. et al. The climate hazards infrared precipitation with stations—A new environmental record for monitoring extremes. *Sci. data* **2**(1), 1–21 (2015). 10.1038/sdata.2015.66 PMC4672685 26646728

[41] DiMiceli, C., Carroll, M., Sohlberg, R., Huang, C. Hansen, M. & Townshend, J. Annual global automated MODIS vegetation continuous fields (MOD44B) at 250 m spatial resolution for data years beginning day 65. (2017).

[42] Esri, E. *ArcMap 10.2* (Redlands, 2014).

[43] Kyale, D. M. *Assessing spatial and temporal patterns of human-caused elephant mortality in Tsavo East National Park, Kenya* (Miami University, 2006).

[44] Abatzoglou, J. T., Williams, A. P., Boschetti, L., Zubkova, M. & Kolden, C. A. Global patterns of interannual climate–fire relationships. *Glob. Chang. Biol.* **24**(11), 5164–5175 (2018). 30047195 10.1111/gcb.14405 PMC7134822

[45] Dunham, K. M. National Summary of Aerial Survey Results for Elephant in Zimbabwe 2014. *Great Elephant Census*. **505**, accessed 26 October 2024. https://www.zamsoc.org/s/Oct-2015-GreatElephantCensusZimbabweNationalSummary2015.pdf (2015).

[46] CoreTeam, Rd., Vienna: R foundation for statistical computing, 2014. (2014).

[47] Dormann, C. F. et al. Collinearity: A review of methods to deal with it and a simulation study evaluating their performance. *Ecography* **36**, 27–46 (2013).

[48] Thuiller, W., Georges, D., Engler, R. & Breiner, F. Biomod2: Ensemble platform for species distribution modeling. *R package version* **2**(7), r560 (2013).

[49] Wato, Y. A. et al. Prolonged drought results in starvation of African elephant (Loxodonta Africana). *Biol. Cons.* **203**, 89–96 (2016).

[50] Thuiller, W.,. Georges, D & Engler, R. Biomod2: Ensemble platform for species distribution modeling. R package version 3.0. 3. https.*CRAN. Rproject. org/package= biomod2*, (2013).

[51] Gregorutti, B., Michel, B. & Saint-Pierre, P. Correlation and variable importance in random forests. *Stat. Comput.* **27**(3), 659–678. 10.1007/s11222-016-9646-1 (2017).

[52] Molina-Vacas, G., Muñoz-Mas, R., Martínez-Capel, F., Rodriguez-Teijeiro, J. D. & Fohlic, G. L. Movement patterns of forest elephants (*Loxodonta**cyclotis* matschie, 1900) in the Odzala-Kokoua National Park, Republic of Congo. *Afr. J. Ecol.* **58**, 23–33 (2020).

[53] Phillips, S. J. Dudík, M. & Schapire, R. E. A maximum entropy approach to species distribution modeling. In *Twenty-first international conference on Machine learning - ICML ’04*, Banff, Alberta, Canada. pp. 83 (ACM Press, 2004). 10.1145/1015330.1015412.

[54] Phillips, S. J. & Dudík, M. Modeling of species distributions with Maxent: New extensions and a comprehensive evaluation. *Ecography* **31**, 161–175 (2008).

[55] Allouche, O., Tsoar, A. & Kadmon, R. Assessing the accuracy of species distribution models: Prevalence, kappa and the true skill statistic (TSS). *J. Appl. Ecol.* **43**(6), 1223–1232. 10.1111/j.1365-2664.2006.01214.x (2006).

[56] Thuiller, W., Georges, D., Engler, R. and Breiner, F. *Ensemble platform for species distribution modeling*. R Package Version: 3.1–64, (2014).

[57] Mumby, H. S., Courtiol, A., Mar, K. U. & Lummaa, V. Climatic variation and age-specific survival in Asian elephants from Myanmar. *Ecology* **94**, 1131–1141 (2013). 23858653 10.1890/12-0834.1

[58] Loveridge, A. J. et al. Bells, bomas and beefsteak: Complex patterns of human-predator conflict at the wildlife-agropastoral interface in Zimbabwe. *PeerJ* **5**, e2898 (2017). 28149682 10.7717/peerj.2898 PMC5267574

[59] Corfield, T. Elephant mortality in Tsavo National Park, Kenya. *Afr. J. Ecol.* **11**, 339–368 (1973).

[60] Conybeare, A. & Haynes, G. Observations on elephant mortality and bones in waterholes. *Quatenary Res.* **22**, 189–200 (1984).

[61] Ramey, E. M., Ramey, R. R., Brown, L. M. & Kelley, S. T. Desert-dwelling African elephants (*Loxodonta**africana*) in Namibia dig wells to purify drinking water. *Pachyderm* **53**, 66–72 (2013).

[62] Wall, J., Douglas-Hamilton, I. & Vollrath, F. Elephants avoid costly mountaineering. *Curr. Biol.* **16**, R527–R529 (2006). 16860724 10.1016/j.cub.2006.06.049

[63] Roever, C. L., Van Aarde, R. J. & Leggett, K. Functional connectivity within conservation networks: Delineating corridors for African elephants. *Biol. Cons.* **157**, 128–135 (2013).

[64] Veerman, J., Kumar, A. & Mishra, D. R. Exceptional landscape-wide cyanobacteria bloom in Okavango Delta, Botswana in 2020 coincided with a mass elephant die-off event. *Harmful Algae* **111**, 102145. 10.1016/j.hal.2021.102145 (2022). 35016759 10.1016/j.hal.2021.102145

[65] D’Araujo and S. R. The relationship between body and environmental temperatures in savanna elephants, *Loxodonta africana*. 2015.

[66] Brahmbhatt, D. P. et al. Contacts between domestic livestock and wildlife at the Kruger National Park Interface of the Republic of South Africa. *Prev. Vet. Med.* **103**, 16–21 (2012). 21907434 10.1016/j.prevetmed.2011.08.003

[67] Vandewalle, M. E. & Alexander, K. A., *Guns, ivory and disease: Past influences on the present status of Botswana’s elephants and their habitats*.In *Elephants and Savanna Woodland Ecosystems*. 91 (2014).

[68] Kyale, D. M., Ngene, S. M. & Maingi, J. Biophysical and human factors determine the distribution of poached elephants in Tsavo East National Park, Kenya. *Pachyderm* **49**(1), 48–60 (2011).

